# ROS inhibit autophagy by downregulating ULK1 mediated by the phosphorylation of p53 in selenite-treated NB4 cells

**DOI:** 10.1038/cddis.2014.506

**Published:** 2014-11-27

**Authors:** Y Ci, K Shi, J An, Y Yang, K Hui, P Wu, L Shi, C Xu

**Affiliations:** 1 State Key Laboratory of Medical Molecular Biology, Institute of Basic Medical Sciences and School of Basic Medicine, Department of Biochemistry and Molecular Biology, Peking Union Medical College and Chinese Academy of Medical Sciences, Beijing, China

## Abstract

Reactive oxygen species (ROS) have an important role in regulating various cellular processes. Our previous study confirmed that selenite, an anti-tumour agent, triggered apoptosis through the production of ROS in multiple types of cancer cells. In this study, we discovered that ROS also inhibited protective autophagy by decreasing the expression of ULK1, an initiator of autophagy, in selenite-treated NB4 cells. Further experiments demonstrated that p-p53 (S392), a phosphorylation event promoted by p70S6K, bound to the promoter of ULK1 and modulated its expression. Experiments in a mouse tumour model with NB4 cells provided *in vivo* confirmation of the alterations in the p70S6K/p53/ULK1 axis. Collectively, our results show that ROS inhibited autophagy by downregulating the p70S6K/p53/ULK1 axis in selenite-treated NB4 cells.

Autophagy is an important homeostatic process that degrades cellular components through lysosome.^1, 2^ Autophagy was once considered as a form of programmed cell death.^3^ However, increasing evidence indicates that autophagy protects cells through the degradation of damaged organelles. Therefore it appears that the relationship between autophagy and cell death is complex and attractive.^4^ Although autophagy is crucial to determine the cell fate, the detailed mechanisms remain unclear.^5^ Data from multiple sources indicate that reactive oxygen species (ROS) have an important role in the induction of autophagy.

ROS, known as multifunctional small reactive molecules, are involved in various processes and regulate cell growth, differentiation, inflammation and immune response. Emerging evidence indicates that ROS may also regulate autophagy through multiple signalling pathways, such as c-Jun N-terminal kinases (JNK), Akt-mTOR (mammalian target of rapamycin)and AMP-activated protein kinase (AMPK).^6, 7^ However, the exact mechanisms of this process require further investigation.

Selenium is an indispensable trace element in humans, while supra-nutritional doses of selenite have been reported to regulate apoptosis and autophagy in tumour cells through various pathways.^8, 9, 10, 11^ Our previous work showed that selenite induced apoptosis and inhibited autophagy in the leukaemia cell line NB4.^9^ Evidence demonstrates that ROS induced by selenite are involved in tumour cell apoptosis.^12^ However, little is known about the relationship between selenite-induced ROS and autophagy. In our previous cDNA microarray analysis, several autophagy-related genes, including Unc-51-like kinase-1 (ULK1), varied at the transcriptional level upon treatment with a supra-nutritional dose of selenite.^13^

ULK1, which is known to be an initiator of autophagy, can be phosphorylated by upstream mTOR and AMPK and then transduce those signals to downstream mediators to regulate autophagy.^14, 15, 16, 17, 18^ In addition to regulation by phosphorylation, ULK1 can also be regulated by p53 at the transcriptional level.^19^ A recent study has also shown that ROS may induce autophagy through ULK1.^20^ Interestingly, we found that ROS inhibited autophagy by downregulating the expression of ULK1 in selenite-induced NB4 cells.

In this report, we found that selenite-induced ROS inhibited autophagy and promoted apoptosis in NB4 cells. Further studies showed that the 70-kDa ribosomal S6 kinase (p70S6K)/p53/ULK1 pathway was involved in this process. Experiments in mouse xenograft tumour model derived from NB4 cells confirmed these results *in vivo*.

## Results

### Selenite-induced ROS inhibits autophagy by increasing ROS in NB4 cells

**Figure 1:**
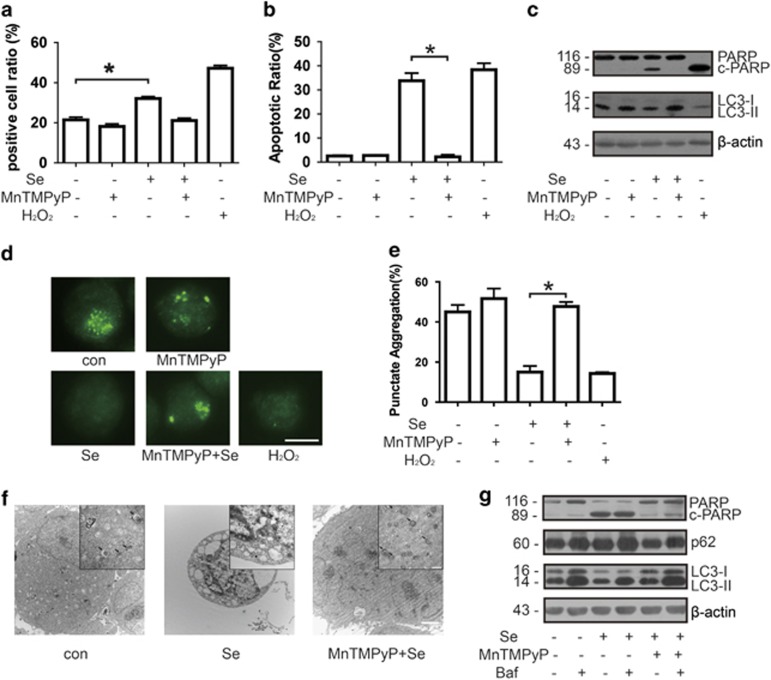
Selenite-induced ROS inhibited autophagy and promoted apoptosis. (**a**) Selenite induced the production of ROS. After treatment with 10 *μ*M MnTMPyP and/or 20 *μ*M selenite, cells were labelled with DCF in the dark, and then the percentage of positive cells was measured by flow cytometry. The data are shown as means±S.D. (*n*=3),*P<0.05. (**b**) ROS induced apoptosis in NB4 cells. Cells were pretreated with 10 *μ*M MnTMPyP for 1 h and then exposed to 20 *μ*M selenite for another 24 h. Cells were treated with 100 *μ*M H_2_O_2_ as a positive control. The percentage of apoptotic cells was measured using flow cytometry. The data are shown as means±S.D. (*n*=3), *P<0.05. (**c**) Selenite-induced ROS inhibited autophagy and promoted apoptosis in NB4 cells. Cells were pretreated with 10 *μ*M MnTMPyP for 1 h and then exposed to 20 *μ*M selenite for another 24 h. Cells were treated with 100 *μ*M H_2_O_2_ as a positive control. c-PARP and LC3-II were detected by western blotting. (**d** and **e**) ROS decreased the percentage of NB4 cells containing LC3 puncta. Cells were pretreated with 10 *μ*M MnTMPyP for 1 h and then exposed to 20 *μ*M selenite for another 24 h. Cells were treated with 100 *μ*M H_2_O_2_ as a positive control. Cells were labelled with anti-LC3 antibody and a DyLight 488-conjugated secondary antibody. The percentage of cells containing more than eight LC3 puncta was calculated. The images were visualised using a Zeiss microscope (Oberkochen, Germany). Bar: 10 *μ*m. Values are shown as means±S.D. (*n*=3), *P<0.05. (**f**) Electron microscopy indicated the alteration of autophagy. After treatment with 10 *μ*M MnTMPyP and 20 *μ*M selenite, cells were submitted to detection by an electron microscope (JEOL,Tokyo, Japan). The arrows point to autophagosomes. The scale bar in the larger image represents 2 *μ*m and that in the smaller image represents 500 nm. (**g**) The effects of ROS to autophagic flux. Cells were pretreated with 10 *μ*M MnTMPyP and/or 5 *μ*M bafilomycin for 1 h and then exposed to 20 *μ*M selenite for another 24 h. p62 and LC3-II were detected by western blotting

Our previous studies indicated that the production of ROS was increased by selenite treatment in several cancer cell lines, including NB4 cells.^12^ Here we measured ROS of NB4 cells with dichlorofluorescein diacetate (DCF) after treatment with selenite. H_2_O_2_ (100 *μ*M) was used as positive control in the experiment. MnTMPyP, a ROS scavenger, was also used to check if it could reverse the effect of selenite. After labelling with DCF, flow cytometry was used to analyse the ROS level of NB4 cells, and results showed that selenite induced the increase of ROS. MnTMPyP could mostly reverse the effect by selenite (Figure 1a). Consistent with our previous results, selenite induces significant apoptosis of NB4 cells. Although MnTMPyP could protect NB4 cells from selenite-induced apoptosis (Figure 1b and Supplementary Figure S1). We further checked poly (ADP-ribose) polymerase (PARP), a marker of apoptosis, by western blotting. Data showed that c-PARP (cleaved form) is increased upon selenite treatment, confirming the result from flow cytometry. Again MnTMPyP reverse the effect in NB4 cells (Figure 1c, top panel).

However, whether ROS could affect autophagy has remained unclear. To explore the relationship between ROS and autophagy, we pretreated cells with MnTMPyP to reduce ROS for 1 h followed by treatment of 20 *μ*M selenite for 24 h. By calculating the percentage of cells containing microtubule-associated protein light chain 3 (LC3) puncta, we found that like H_2_O_2_, a classic ROS, selenite treatment greatly inhibit autophagy. MnTMPyP could reverse the anti-autophagy effect of selenite (Figures 1d and e). Consistent with the above results, combined treatment with MnTMPyP and selenite in NB4 cells also led to the release of selenite effects on autophagy at the protein levels in NB4 cells. LC3-II was increased in the presence of MnTMPyP and selenite, implying that MnTMPyP antagonised the anti-autophagic effect of selenite (Figure 1c). Furthermore, after the treatment of selenite and/or MnTMPyP, we performed electron microscopy to observe autophagosome, and the findings confirmed the above results (Figure 1f). To further verify selenite effect of anti-autophagy, we next measured autophagic flux after bafilomycin treatment. Cells were pretreated with bafilomycin and/or MnTMPyP and then incubated with selenite for 24 h. Selenite treatment led to the decrease of LC3-II as well as the increase of p62/Sequestosome-1, which is degraded by autophagy, whereas MnTMPyP attenuated its effects. Bafilomycin treatment would further increase the level of p62 and led to the accumulation of LC3-II, suggesting that selenite has its anti-autophagy effects at the initiation step (Figure 1g). To further verify the hypothesis that ROS had an important role in autophagy inhibition and apoptosis induction in NB4 cells treated with selenite, we used another antioxidant, MnTBAP, to measure the level of autophagy and apoptosis. We did the same measurements with MnTBAP and acquired similar results with MnTMPyP (Supplementary Figure S2). Taken together, these results indicate that ROS had an important role in selenite-induced apoptosis and inhibition of autophagy in NB4 cells.

### Selenite-induced ROS inhibited autophagy by downregulating the expression of ULK1

**Figure 2:**
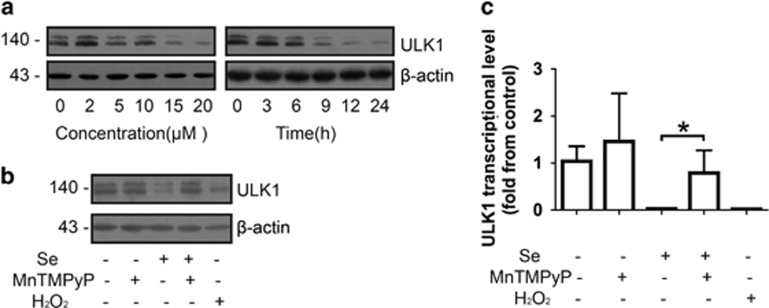
ROS inhibited autophagy by decreasing the level of ULK1. (**a**) ULK1 decreased in a dose- and time-dependent manner. After cells were treated with the indicated doses of selenite for 24 h or 20 *μ*M selenite for the indicated periods of time, the level of ULK1 was detected by western blotting. (**b** and **c**) ROS also downregulate the expression of ULK1. Cells were pretreated with 10 *μ*M MnTMPyP for 1 h and then exposed to 20 *μ*M selenite for another 24 h. Cells were treated with 100 *μ*M H_2_O_2_ as a positive control. The level of ULK1 at the protein and mRNA level were measured through western blotting and real-time PCR, respectively. The expression of the housekeeping gene *β*-actin was used as a reference for normalisation in real-time PCR

**Figure 3:**
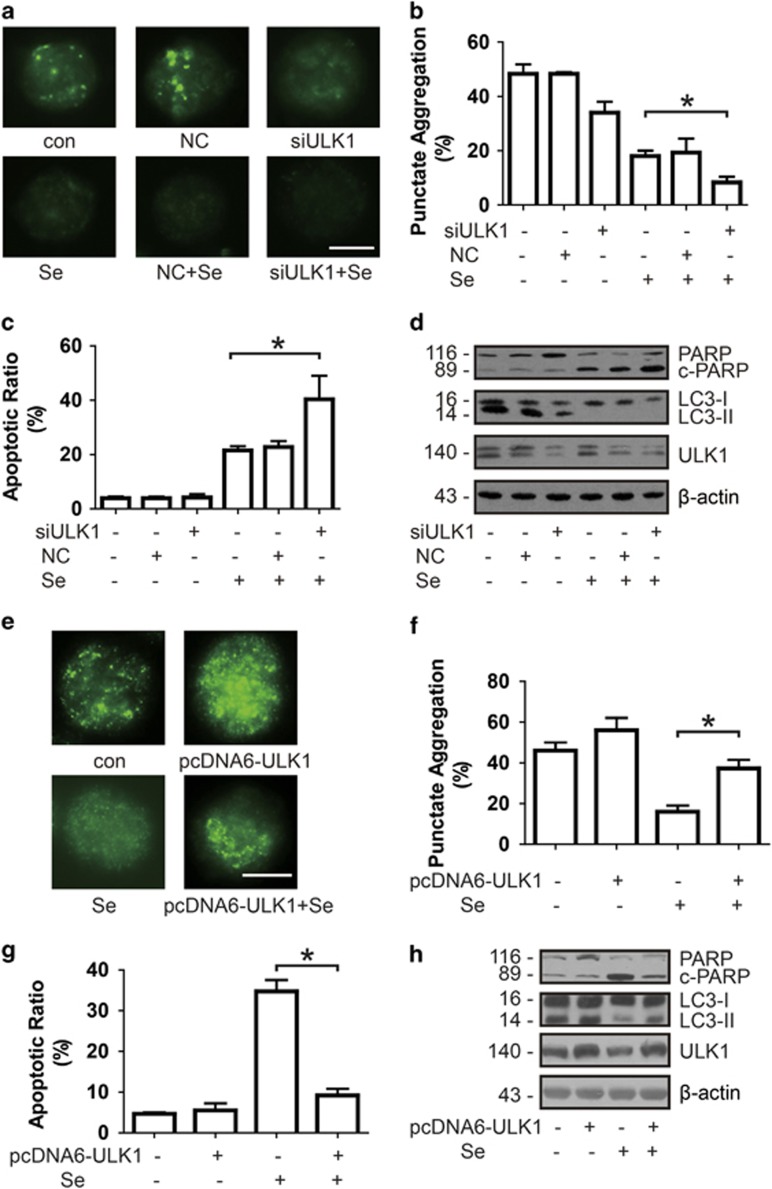
ULK1 initiated autophagy and protected NB4 cells from apoptosis. (**a** and **b**) ULK1 induced autophagy. Cells were transfected with siRNA targeting ULK1 and then indirectly labelled with anti-LC3 antibody. The images were visualised using a Zeiss microscope. Bar: 10 *μ*m. Values are shown as means±S.D. (*n*=3), *P<0.05. (**c**) The deletion of ULK1 promoted apoptosis in NB4 cells. The percentage of apoptotic cells was measured using flow cytometry after NB4 cells were transfected with siRNA targeting ULK1. The data are shown as means±S.D. (*n*=3), *P<0.05. (**d**) The downregulation of ULK1 inhibited autophagy consistent with the increase of apoptosis. (**e** and **f**) Western blotting analysis was performed to detect the levels of LC3-II and c-PARP after depletion of ULK1. The overexpression of ULK1 attenuated the switch from autophagy to apoptosis. After transfection with pcDNA-ULK1 plasmid, cells were incubated in 20 *μ*M selenite and then indirectly labelled with anti-LC3 antibody. The images were visualised using a Zeiss microscope. Bar: 10 *μ*m. Values are shown as means±S.D. (*n*=3), *P<0.05. (**g**) Furthermore, flow cytometry was performed to measure the percentage of apoptosis cells. The data are shown as means±S.D. (*n*=3), *P<0.05. (**h**) Western blotting was also used to measure the level of LC3-II and c-PARP

To explore the detailed mechanisms of the decrease in autophagy mediated by selenite, we screened for key regulators of the autophagy pathway, which may be involved in selenite-induced autophagy inhibition. In our previous study, we screened several hundred autophagy-related genes by cDNA microarray to characterise the changes on transcriptional level in NB4 cells upon selenite treatment. ULK1, an initiator of autophagy, was downregulated at the mRNA level.^13^ We measured the expression of ULK1 by western blotting and found that selenite treatment led to a decrease of ULK1 in a dose- and time-dependent manner (Figure 2a). Similarly, H_2_O_2_ treatment also resulted in a decrease of ULK1. Again, MnTMPyP treatment reversed the effect of selenite at the protein and mRNA level (Figures 2b and c). Furthermore, we examined the effects of ULK1 on autophagy and apoptosis. After ULK1 was knocked down by siRNA, the number of LC3-positive cells decreased (Figures 3a and b), whereas the percentage of apoptotic cells increased (Figure 3c and Supplementary Figure S3a). Additionally, the cleavage of PARP increased and LC3-II declined as predicted (Figure 3d). When ULK1 was overexpressed by transfected ULK1 plasmid, the anti-autophagy and pro-apoptosis effects of selenite were attenuated (Figures 3e–h and Supplementary Figure S3b). These results indicate that the effects of ROS on the induction of apoptosis and the inhibition of autophagy by selenite were mediated by the downregulation of ULK1.

### The expression of ULK1 was regulated by p-p53 (Ser392)

**Figure 4:**
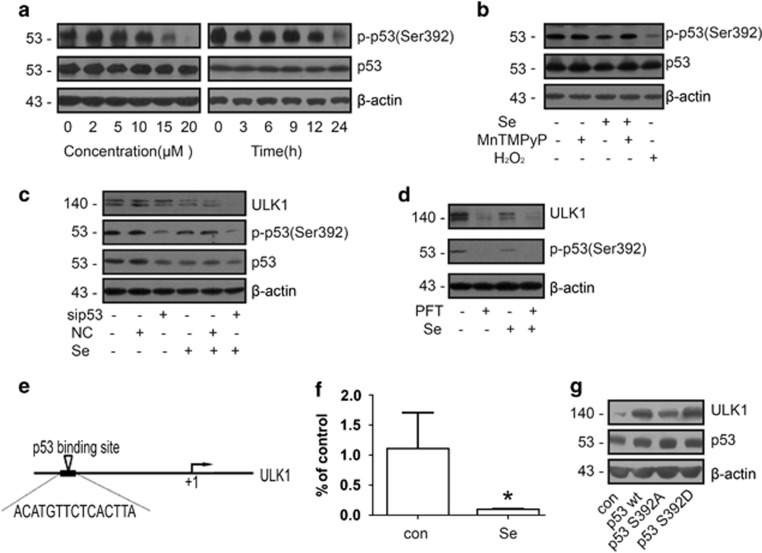
p-p53 (Ser392) promoted the expression of ULK1. (**a**) After exposure to selenite, p-p53 (Ser392) decreased in a dose- and time-dependent manner. After cells were treated with the indicated doses of selenite for 24 h or 20 *μ*M selenite for the indicated periods of time, the phosphorylation of p53 at Ser392 was detected by western blotting. (**b**) Selenite-induced ROS inhibited the phosphorylation of p53 at Ser392. Cells were pretreated with 10 *μ*M MnTMPyP for 1 h and then exposed to 20 *μ*M selenite for another 24 h. Cells were treated with 100 *μ*M H_2_O_2_ as a positive control. p53 and p-p53 (Ser392) were detected by western blotting. (**c**) p-p53 (Ser392) upregulated the expression of ULK1. After transfection of siRNA targeting p53, the levels of p53, p-p53 (Ser392) and ULK1 were measured by western blotting. (**d**) p-p53 (Ser392) increased the expression of ULK1. After cells were pretreated with Pifithrin-α for 1 h and then treated with selenite for another 24 h, p-p53 (Ser392) and ULK1 were detected by western blotting. (**e**) The predicted binding site of p53 at the promoter of ULK1. (**f**) p-p53 (Ser392) directly bound to the promoter of ULK1. After treatment with 20 *μ*M selenite for 24 h, ChIP was performed with an antibody against p-p53 (Ser392), and then real-time PCR was performed immediately. *β*-Actin was used as an internal control for the sample input. (**g**) The phosphorylation of p53 at Ser392 regulated the expression of ULK1. Cells were transfected with wild-type p53, p53 S392A and p53 S392D plasmids, and then western blotting was performed to measure the expression of ULK1

It has been shown that p53 regulates ULK1 transcription.^19^ So we first tested whether the p53 protein level or phosphorylation status changes in NB4 cells after selenite treatment. Although the overall amount of p53 remained the same, its phosphorylation at Ser392 was significantly decreased (Figure 4a). Similarly, H_2_O_2_ significantly inhibited the phosphorylation of p53 at Ser392. However, combined treatment with MnTMPyP and selenite reversed the effects of selenite on the phosphorylation of p53 at Ser392 (Figure 4b). To explore the role of p-p53 in the regulation of ULK1 transcription in selenite-treated NB4 cells, we knocked down p53 by RNA interference and found that the expression of ULK1 was decreased correspondingly (Figure 4c). Moreover, pretreatment of NB4 cells with Pifithrin-α, an inhibitor of p53, further decreased the expression of ULK1 (Figure 4d). A previous study predicted that p53 could bind to the promoter of ULK1 by EMSA.^19^ We analysed the ULK1 promoter region by using the JASPAR software (Copenhagen, Denmark) and found several potential p53-binding sites, one of which is located approximately 2.1 kb upstream of the ULK1 transcriptional start site (Figure 4e). We used an antibody that specifically recognises p-p53 (Ser392) to perform chromatin immunoprecipitation. The result showed that p-p53 (Ser392) could bind to this site in ULK1 promoter region, and selenite effectively attenuated this interaction (Figure 4f), suggesting that p-p53 is an upstream regulator of ULK1. To further confirm that it was the phosphorylation at Ser392 of p53 that regulated the expression of ULK1, we mutated Ser392 of p53 to Ala or Asp, respectively. After transfection of NB4 cells with wild-type or mutant p53 (S392A or S392D) plasmids, western blotting was performed to measure the level of ULK1. Data showed that p53 S392A could not increase the expression of ULK1 as wild-type p53, whereas p53 S392D induced a higher expression of ULK1 comparing to wild-type p53 (Figure 4g). Thus p-p53 is directly responsible for the downregulation of ULK1 expression in selenite-treated NB4 cells.

### p70S6K phosphorylated p53 at Ser392

**Figure 5:**
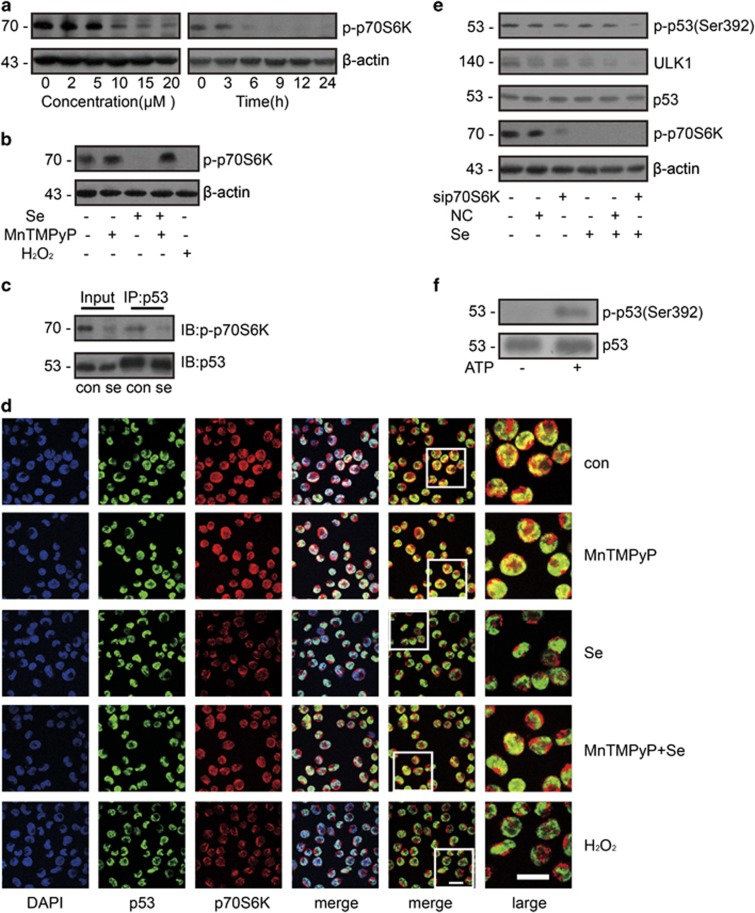
p-p70S6K phosphorylated p53 at Ser392. (**a**) p-p70S6K decreased in a time- and dose-dependent manner upon treatment with selenite. After exposure to selenite, p-p70S6K decreased in a time- and dose-dependent manner. After cells were treated with the indicated doses of selenite for 24 h or 20 *μ*M selenite for the indicated periods of time, p-p70S6K was detected by western blotting. (**b**) ROS decreased the activity of p70S6K. Cells were pretreated with 10 *μ*M MnTMPyP for 1 h and then exposed to 20 *μ*M selenite for another 24 h. Cells were treated with 100 *μ*M H_2_O_2_ as a positive control. p-p70S6K was detected by western blotting. (**c**) p-p70S6K interacted with p53. After treatment with 20 *μ*M selenite for 24 h, whole cell lysates were extracted, and immunoprecipitation was performed. The interaction between p-p70S6K and p53 was visualised by western blotting. (**d**) p70S6K co-localised with p53. After cells were exposed to the indicated treatment, p53 and p70S6K were labelled with primary antibodies and fluorescein isothiocyanate- or Cy3-conjugated secondary antibodies. The images were visualised with a confocal microscope. Bar: 20 *μ*m. (**e**) p70S6K increased the phosphorylation of p53 and the expression of ULK1. After transfection of p70S6K siRNA, alterations of p-p70S6K, p53, p-p53 (Ser392) and ULK1 were detected by western blotting. (**f**) p70S6K directly phosphorylated p53 at Ser392. Recombinant p53 protein and p70S6K recombinant protein was co-incubated in Kinase Solution with/without ATP at 37 °C. The level of p-p53 (Ser392) was measured by western blotting with p-p53 (Ser392)-specific antibody

To identify upstream regulator of p53, we screened a few kinases by immnoprecipitation using p53 antibody and found p70S6K might be a promising candidate. In another study, p70S6K was shown to be an upstream regulator of p53.^21^ We first checked whether p70S6K changes after selenite treatment and found that phosphorylation of p70S6K decreased in a dose- and time-dependent manner (Figure 5a). Moreover, the phosphorylation of p70S6K was reversed by ROS scavenging (Figure 5b). In the immunoprecipitation experiment, p-p70S6K interacted with p53 and that this interaction was attenuated by selenite (Figure 5c). Furthermore, immunofluorescence staining showed that p53 and p70S6K were significantly co-localised and that selenite disrupted their co-localisation (Figure 5d). As expected, in the presence of MnTMPyP and selenite, the disruption of the interaction between p53 and p70S6K was inhibited, whereas H_2_O_2_ also decreased the co-localisation of p53 and p70S6K. To further confirm the effect of p70S6K on phosphorylation of p53 (Ser392), we knocked down p70S6K and found that p53 (Ser392) phosphorylation was downregulated and thus decrease ULK1 expression (Figure 5e). To confirm that p70S6K could phosphorylate p53, we performed a kinase assay *in vitro*, suggesting that p70S6K directly phosphorylated p53 at Ser392 (Figure 5f). These results demonstrated that p70S6K directly phosphorylates p53 to regulate its function.

### Selenite regulated autophagy and apoptosis through the p70S6K/p53/ULK1 pathway *in vivo*

**Figure 6:**
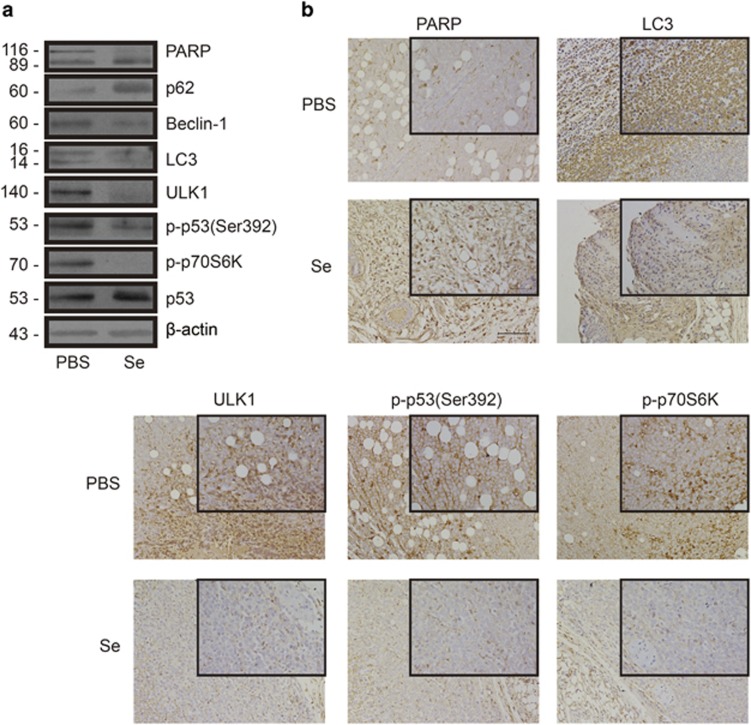
p-p70S6K, p-p53 (Ser392) and ULK1 were regulated by selenite *in vivo*. (**a**) The p70S6K/p53/ULK1 pathway was altered by selenite *in vivo*. Whole-tissue lysates were extracted, and the levels of p-p70S6K, p-p53 (Ser392), p53, ULK1, LC3-II and c-PARP were detected by western blotting. (**b**) The p70S6K/p53/ULK1 pathway was regulated by selenite *in vivo*. After immunohistochemical staining, the images were captured with a Nikon microscope (Tokyo, Japan). The scale bar in the larger image represents 100 *μ*m and that in the smaller image represents 50 *μ*m

After identifying the p70S6K/p53/ULK1 pathway in NB4 cells, we next wanted to test whether the same pathway existed *in vivo*. Our lab previously established a xenograft tumour model in nude mice. Tumours became visible at 14 days after NB4 cells were injected into mice. Upon tumour detection, mice were randomly divided into two groups (six mice per group). These mice were injected with PBS or selenite (3 mg, every 2 days, i.p.) for 18 days. As previously reported, selenite treatment dramatically inhibited tumour growth by inducing apoptosis.^22^ We extracted tissue lysates from tumour cells and performed western blottings. The levels of p-p70S6K, p-p53 (Ser392), ULK1, LC3 and C-PARP were altered in the same manner as they did in NB4 cells (Figure 6a). Immunohistochemical staining confirmed the above results (Figure 6b). In summary, selenite inhibited autophagy through the p70S6K/p53/ULK1 pathway *in vivo*, and apoptosis was correspondingly induced.

## Discussion

Because autophagy is related to fate determination in cancer cells, understanding the relationship between autophagy and apoptosis is critical for the development of cancer therapies. Our previous study showed that autophagy protects NB4 cells from apoptosis. In the present work, we first found that selenite-induced ROS inhibited autophagy by decreasing the expression of ULK1, which was transcriptionally regulated by p53. Moreover, we identified p70S6K as an upstream regulator of p53; the phosphorylation of p70S6K decreased upon selenite treatment, thereby inhibiting the phosphorylation of p53 at Ser392. Together, our data indicate that selenite-induced ROS inhibited autophagy through the p70S6K/p53/ULK1 pathway and promoted cell apoptosis at the same time.

Because autophagy is known to often co-occur with cell death, the relationship between autophagy and cell death has been extensively investigated.^23, 24, 25, 26^ Autophagy was previously known as type II programmed cell death, also termed ‘autophagic cell death,' and was found to be associated with the accumulation of autophagosomes in cells.^3^ A recent study indicated that, when autophagy was inhibited, apoptosis was increased adversely in chronic myeloid leukaemia.^27^ Consistent with that report, we also observed that selenite promoted the switch from protective autophagy to apoptosis in NB4 cells previously,^9^ and here we determined that the decrease of autophagy in NB4 cells was regulated by the production of ROS.

Previous studies indicated that ROS are mutagenic and may thus promote cancer,^28^ but a recent study found that ROS may inhibit cancer development.^29^ Several studies also reported that ROS functioned as key intermediates of apoptosis that was induced by multiple agents.^30^ Consistently, our previous studies also indicated that selenite induced a rapid increase in ROS in NB4 cells. Meanwhile, selenite-induced ROS promoted apoptosis through multiple pathways, such as JNK/ATF2 and Ras homolog gene family, member A/Ser/Thr Rho kinase 1.^12, 22^ Additionally, mitochondrial ROS are critical for autophagy.^31^ It is generally accepted that ROS induce autophagy and that this process is mediated by various signalling cascades.^32, 33^ For example, ROS inhibit mTOR by activating AMPK and inhibiting AKT, leading to autophagy-dependent cell death.^34^ However, considering that some tumour cells take advantage of protective autophagy to survive, inhibition of protective autophagy may be a potential strategy for cancer therapy. When we treated NB4 cells with selenite, in contrast to other cell types, we observed that increased levels of ROS inhibited autophagy. Similarly, H_2_O_2_, a classic ROS used as a positive control in the present study, also inhibited autophagy in NB4 cells. Meanwhile, apoptosis in NB4 cells increased upon treatment with selenite or H_2_O_2_. This is a good example of the antagonism between autophagy and apoptosis in tumour cells: protective autophagy switched to apoptosis under selenite treatment. We next explored the mechanisms of the anti-autophagic effects of ROS and found that p70S6K acted as a messenger between ROS and autophagy.

The 70-kDa ribosomal S6 kinase (p70S6K) is a serine/threonine kinase, and its target substrate is the S6 ribosomal protein.^35^ Previous evidence has shown that p70S6K was activated by an increase in ROS.^36^ A more recent study also demonstrated that phospho-p70S6K inhibited autophagy, although the underlying mechanism remained unknown.^37^ However, our results showed that inactivation of p70S6K led to the downregulation of autophagy, which was mediated by selenite-induced ROS. Further data indicated that phospho-p70S6K phosphorylated p53 at Ser392 directly.

p53 is a well-known tumour suppressor involved in autophagy and apoptosis. In recent years, an increasing number of studies has indicated that p53 has dual roles in the regulation of autophagy.^38^ Nuclear p53 has been shown to promote autophagy through the transcriptional regulation of downstream genes involved in the autophagic pathway, such as AMPK-*β*1/2, phosphatase and tensin homolog, tuberous sclerosis 1/2, Sesn1/2 and DRAM (dynamic random-access memory,^39, 40^ whereas cytoplasmic p53 inhibits autophagy.^38^ In our system, although total p53 was not altered after NB4 cells were treated with selenite, phospho-p53 (Ser392) clearly decreased. Further experiments indicated that p53 was phosphorylated by p-p70S6K at Ser392. Therefore the downregulation of p-p70S6K after selenite treatment inhibited the phosphorylation of p53 at Ser392. Extensive investigations into the relationship between p-p53 and autophagy showed that phospho-p53 (Ser392) might be one of the upstream transcriptional regulators of ULK1 to regulate the process of autophagy.

ULK1, as the initial component of the mammalian autophagy pathway, functions in a complex with autophagy-related gene 13 (Atg13), FIP200 (FAK family-interacting protein of 200 kDa) and Atg101,^41, 42, 43, 44, 45^ and its phosphorylation is considered to have an essential role in the regulation of autophagy, which is regulated by AMPK and mTOR. The phosphorylation of ULK1 at different sites may have distinct functions in autophagy,^18^ but the detailed mechanisms of this process remain to be discovered. In our previous cDNA array screen for autophagy-related genes, transcription of ULK1 in NB4 cells significantly decreased after selenite treatment. The current study demonstrates that phospho-p53 (Ser392) binds to the promoter of ULK1, suggesting that p53 may regulate the transcription of ULK1. In another report, p53 was identified as an upstream regulator of ULK1;^19^ our result further implies that the phosphorylation of p53 at Ser392 is important for this regulation.

To confirm the results observed in the NB4 cell line, we used a xenograft tumour model and found that selenite significantly inhibited the growth of tumours derived from NB4 cells in nude mice. Using the same mouse model, we also examined the protein levels of PARP, LC3, p-p70S6K, p-p53 (Ser392) and ULK1. These results were consistent with those from NB4 cells, suggesting that selenite may inhibit autophagy and induce apoptosis *in vivo* through a similar mechanism.

**Figure 7:**
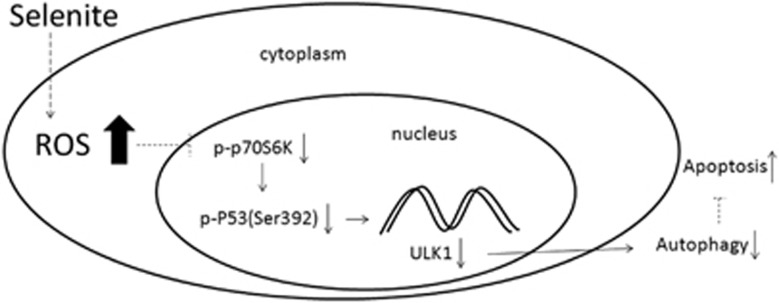
Selenite-induced ROS inhibited the activity of p70S6K, which regulated the phosphorylation of p53 at Ser392. p-p53 (Ser392) acted as a transcription factor to promote the expression of ULK1, an initiator of autophagy, and altered the levels of autophagy and apoptosis

In summary, we showed that selenite treatment resulted in a rapid increase in ROS in NB4 cells and thus induced apoptosis and blocked protective autophagy through the p70S6K/p53/ULK1 pathway (Figure 7). Similar effect was observed in NB4-derived tumour *in vivo*. Some other molecules may also be involved in this process, and further studies are required to reveal the detailed mechanisms.

## Materials and Methods

### Cell culture

NB4 cells were grown at 37 °C with 5% CO_2_ in RPMI 1640 supplemented with 10% FBS, 0.2% sodium bicarbonate, 100 units/ml penicillin and 100 units/ml streptomycin.

### Chemicals and antibodies

Active p70S6K recombinant protein, anti-*β*-actin antibody, bafilomycin and sodium selenite were purchased from Sigma-Aldrich (St Louis, MO, USA). Pifithrin-α and MnTMPyP were purchased from Merck Calbiochem (San Diego, CA, USA). Anti-p53 antibody and MnTBAP was purchased from Santa Cruz Biotechnology (Santa Cruz, CA, USA). Anti-ULK1 and anti-LC3 antibodies (for immunofluorescence) were purchased from Abgent (San Diego, CA, USA). Anti-p-p53 (Ser392) antibody was purchased from Nanjing EnoGene Biotechnology (Nanjing, China). Anti-p70S6K antibody was obtained from Proteintech Group, Inc. (Chicago, IL, USA). The HRP-conjugated anti-mouse (ZB-2305) and anti-rabbit (ZB-2301) antibodies were obtained from ZSGB-BIO (Beijing, China). Anti-p-p70S6K, anti-LC3 and DyLight 488-conjugated anti-rabbit secondary antibody were purchased from Cell Signalling Technology (Danvers, MA, USA). The Cy3-conjugated anti-rabbit (89856) and FITC-conjugated anti-mouse (89750) antibodies were purchased from Jackson ImmunoResearch (West Grove, PA, USA). p53 recombinant protein was obtained from Boston Biochem (Cambridge, MA, USA).

### Western blotting

Cells were collected, washed with PBS and then lysed in a solution (RIPA) containing 20 mM Tris (pH 7.5), 150 mM NaCl, 1 mM EDTA, 1 mM EGTA, 1% Triton X-100, 2.5 mM sodium pyrophosphate, 1 mM *β*-glycerophosphate, 1 mM Na_3_VO_4_ and protease inhibitors (10 *μ*g/ml aprotinin, 1 *μ*g/ml leupeptin and 0.1 mM phenylmethylsulfonyl fluoride). Equal amounts of samples were subjected to electrophoresis on a 10%-15% SDS-PAGE gel and then transferred to nitrocellulose membranes. The membranes were blocked in Tris-buffered saline containing 0.1% Tween-20 (TBST) and 5% non-fat milk for 1 h and incubated with primary antibodies at 4 °C overnight. The membranes were then washed with TBST and incubated with HRP-conjugated secondary antibodies for 1 h at room temperature. After washing, immunoreactive bands were visualised with the SuperSignal chemiluminescent substrate (PerkinElmer, Waltham, MA, USA).

### Flow cytometric assessment of apoptosis

Cells were collected and washed with ice-cold PBS, resuspended in binding buffer containing a suitable amount of Annexin V-FITC and propidium iodide and then incubated for 15 min on ice in the dark. Stained cells were subjected to flow cytometric analysis to detect apoptosis.

### Immunoprecipitation

Approximately 4 × 10^6^ cells were collected, resuspended in RIPA buffer and lysed on ice for 1 h. The lysates were centrifuged at 12 000 r.p.m. for 10 min at 4 °C, and the supernatant was transferred to a new tube. Next, 2 *μ*l of primary antibodies was added to 200 *μ*g protein, and the mixture was gently rotated at 4 °C overnight. The immune complex was captured by protein A+G beads for 3 h at 4 °C and then washed with RIPA buffer three times. The bead mixtures were resuspended in SDS loading buffer and boiled for 5 min.

### Immunofluorescence

Cells were collected and washed with PBS, and a cytospan were then used to adhere the cells onto glass slides. The cells were fixed in ice-cold methanol for 10 min and washed with PBS. The cells were blocked in blocking buffer for 1 h and then incubated with primary antibodies overnight at 4 °C. After washing, the cells were incubated with secondary antibodies at room temperature for 1 h. Images were visualised by confocal microscopy.

### Chromatin immunoprecipitation

A simple ChIP Enzymatic Chromatin IP Kit was purchased from Cell Signaling Technology. Formaldehyde was added to 10 ml of cell suspension to promote protein–chromatin crosslinking, and the crosslinking was stopped by the addition of glycine at a final concentration of 0.125 M. The cells were collected and washed with ice-cold PBS. The pellets were resuspended in buffer A and incubated on ice for 10 min. The nuclear fraction was collected, resuspended in buffer B and then digested with micrococcal nuclease at 37 °C. After the reaction was stopped with 0.5 M EDTA, the samples were centrifuged at 13 000 r.p.m. for 10 min. The pellets were resuspended in buffer B and then incubated with p-p53 (Ser392) antibody overnight at 4 °C. The complex was captured using the ChIP-Grade Protein G Agarose Beads for 3 h at 4 °C. Finally, PCR analysis was performed with primers for the p53 site in the ULK1 promoter.

### Transfections

Cells were collected and resuspended in 200 *μ*l Opti-MEM medium (Gibco, Grand Island, NY, USA). The cell suspension was transferred into electroporation cuvettes, and 200 nM siRNA was added. The cuvettes were then electroporated (a typical capacitance value is 1075 F, with a voltage of 220 V). After 60–80 s, the cuvettes were removed and placed on ice, and the cell suspension was transferred into culture medium.

### Real-time PCR

Total RNA was extracted by Trizol agent (Invitrogen, Carlsbad, CA, USA). Total RNA from each sample was submitted to reverse transcription with 5 × All-In-One RT MasterMix (abmGood, Richmond, BC, Canada). The expression of target gene was normalised to *β*-actin gene expression level. Real-time PCR was performed with UltraSYBR Mixture (With ROX) (CWBIO, Beijing, China) on Bio-Rad IQ5 (Bio-Rad, Hercules, CA, USA).

### ROS measurement

About 1.2 × 10^6^ cells treated with the indicated agents were collected, then washed with serum-free medium and incubated with 5 *μ*M DCFH-DA (Beyotime, Nantong, China) at 37 °C for 20 min. After washing with serum-free medium, cells were submitted to flow cytometric analysis.

### Electron microscopy

Cells were harvested and fixed in 2.5% glutaraldehyde for at least 3 h. The following steps had been mentioned previously.^46^ The images were visualised by transmission electron microscopy.

### Kinase assay

p53 and active p70S6K recombinant protein were added into precooled microcentrifuge tube combined with 20 *μ*l Kinase Solution (a mixture of 5 mM MOPS, pH 7.2, 2.5 mM glycerol 2-phosphate, 5 mM MgCl_2_, 1 mM EGTA and 0.4 mM EDTA, and just prior to use, DTT added to a final concentration of 0.25 mM). Each reaction was initiated with the addition of 5 *μ*l of 250 *μ*M ATP solution, and then the mixture was incubated at 37 °C for 4 h. The mixture was measured by western blotting with antibodies against p53 and p-p53 (Ser392).

### Immunohistochemical staining

The slides were deparaffinised and dehydrated with decreasing concentrations of ethanol. After heating in citrate buffer, the slides were incubated with primary antibodies overnight at 4 °C and then blocked with peroxide. Subsequently, the slides were incubated with secondary antibodies at room temperature for 30 min, treated with DAB and stained with haematoxylin. After dehydration and clarification with xylene, the slides were mounted with mounting medium.

### Statistical analysis

Values are shown as means±S.D. Two-tailed Student's *t*-tests were used for comparisons between two groups, and *P*<0.05 was considered to be significant.

## Abbreviations

AMPK: AMP-activated protein kinase
Atg: autophagy-related gene
DCF: dichlorofluorescein diacetate
JNK: c-Jun N-terminal kinases
LC3: microtubule-associated protein light chain 3
mTOR: mammalian target of rapamycin
p70S6K: 70-kDa ribosomal S6 kinase
PARP: poly (ADP-ribose) polymerase
ROS: reactive oxygen species
ULK1: Unc-51-like kinase-1
