# Supplementary figures and images for: ROS inhibit autophagy by downregulating ULK1 mediated by the phosphorylation of p53 in selenite-treated NB4 cells
Source: Cell Death Dis. 2014 Nov 27;5(11):e1542–. doi: 10.1038/cddis.2014.506 (PMC4260759; doi:10.1038/cddis.2014.506)

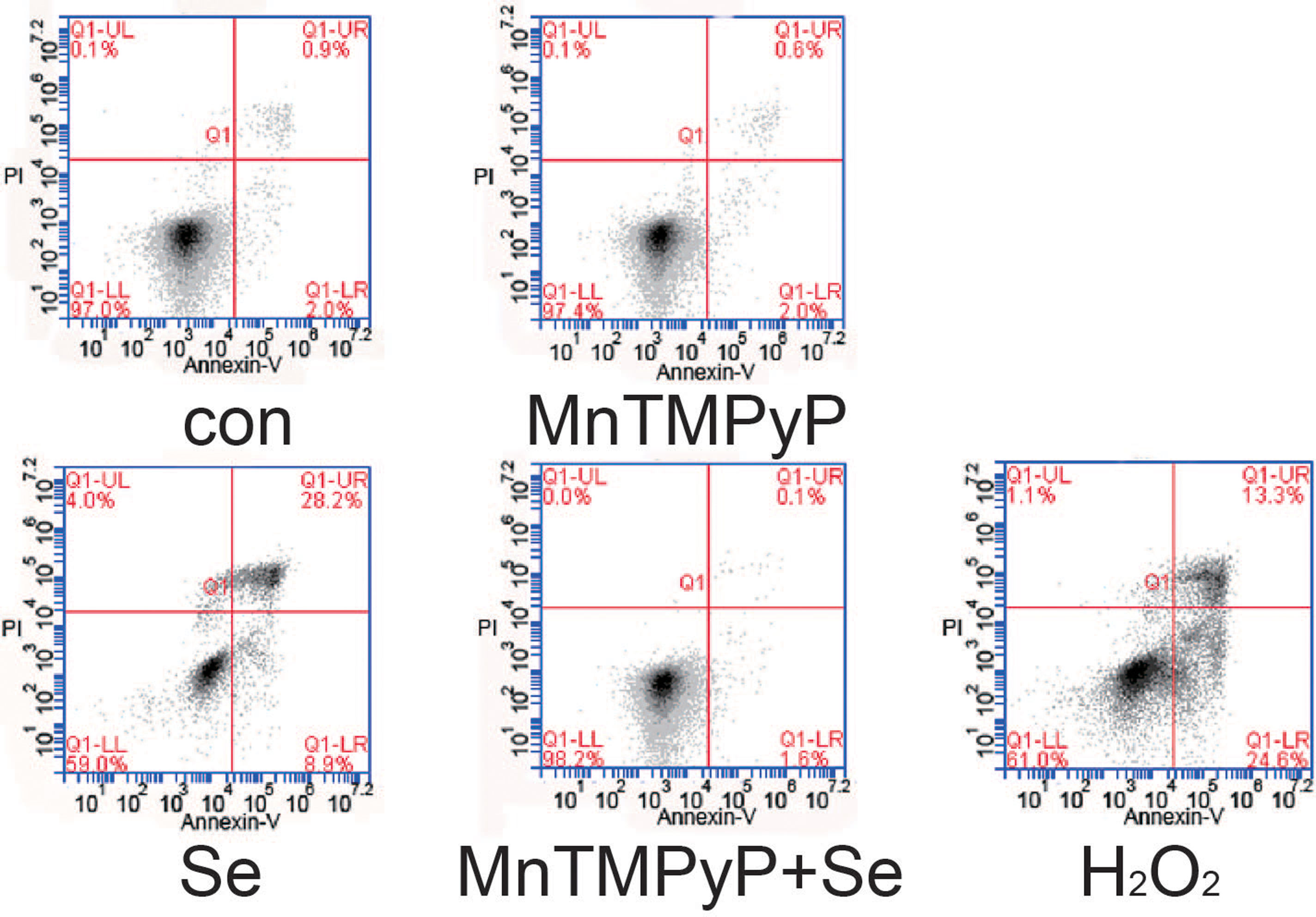

Supplement: Supplementary Figure S1 [file cddis2014506x1.tif]

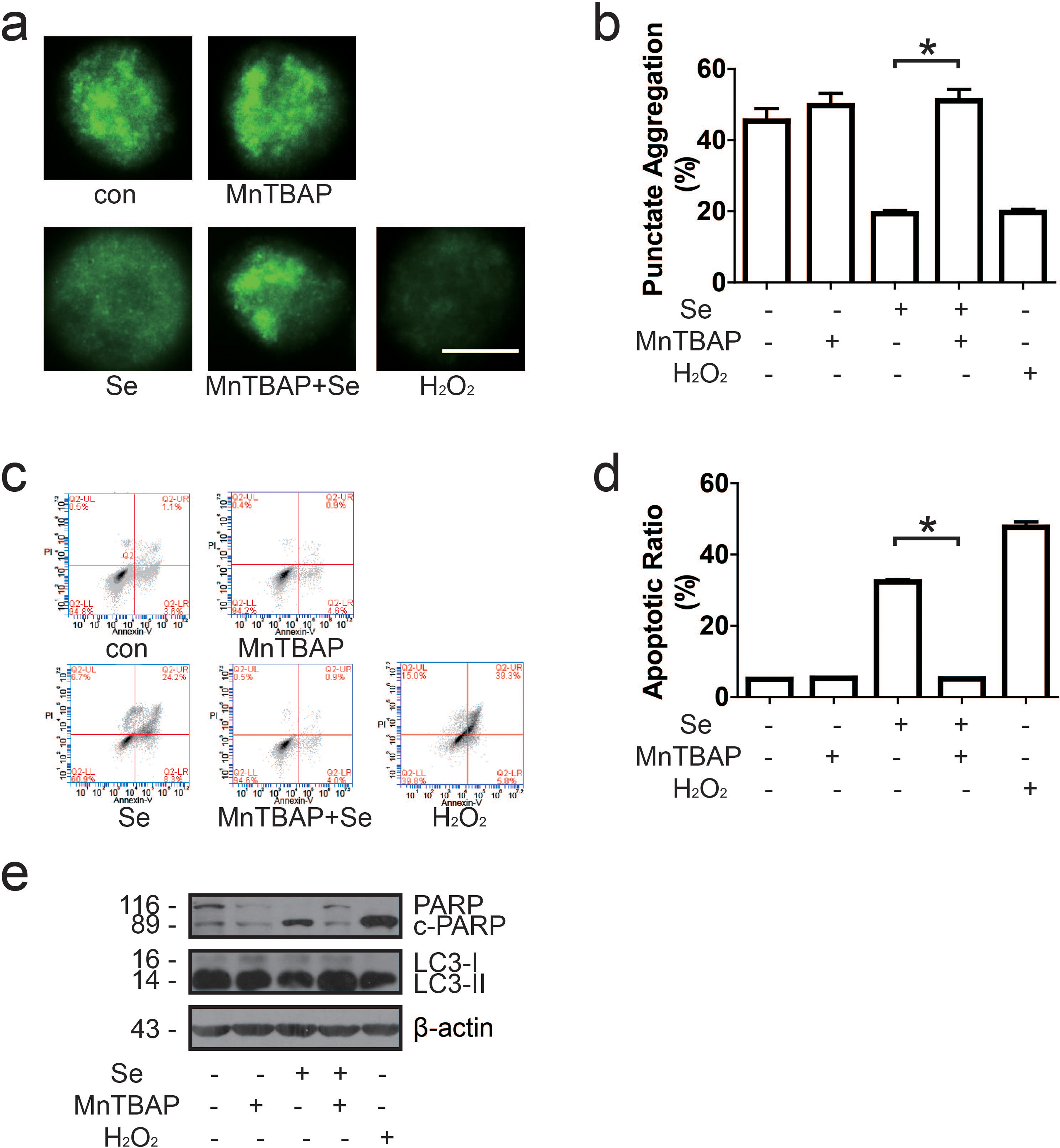

Supplement: Supplementary Figure S2 [file cddis2014506x2.tif]

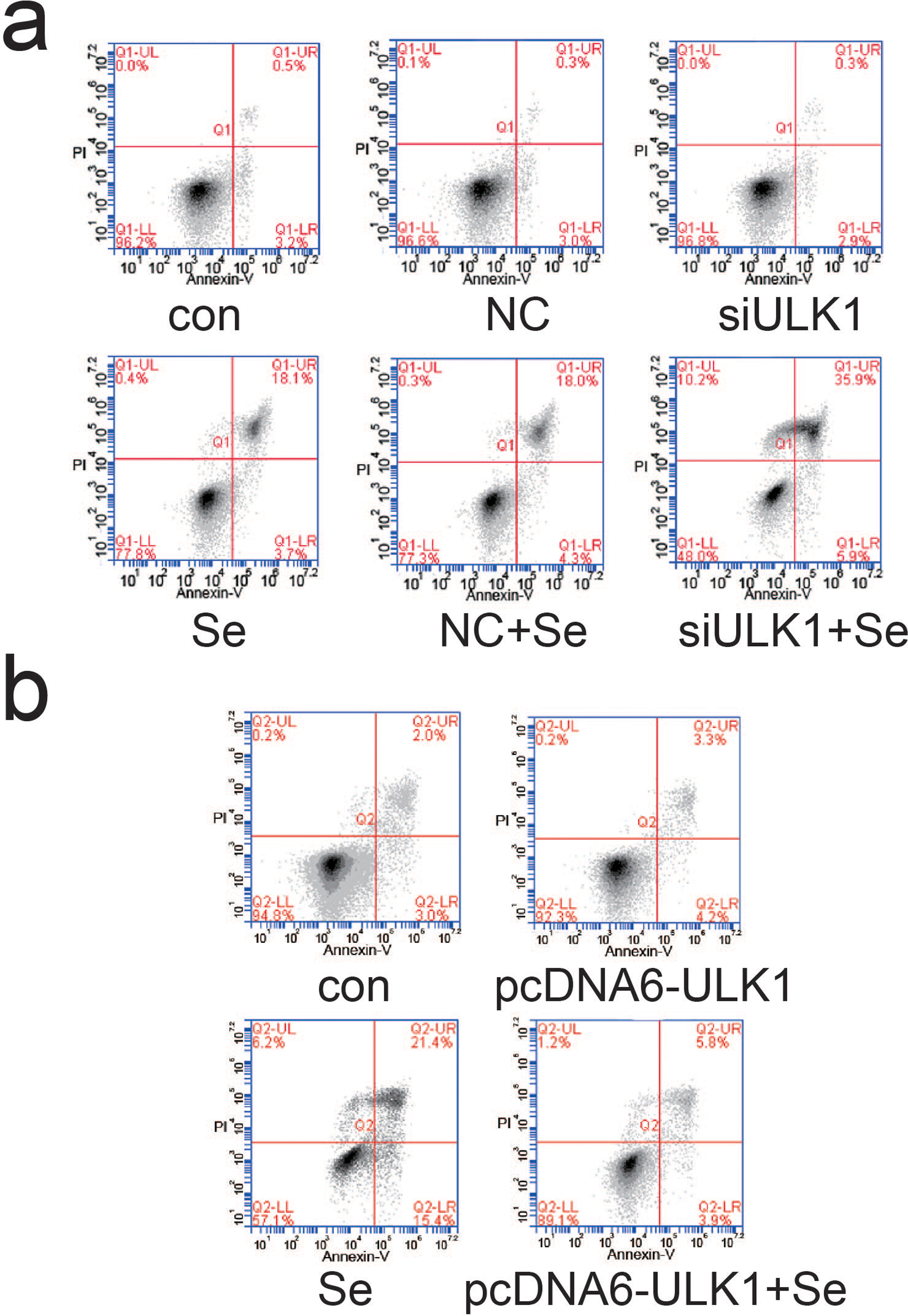

Supplement: Supplementary Figure S3 [file cddis2014506x3.tif]
