# Supplementary material for: ROS inhibit autophagy by downregulating ULK1 mediated by the phosphorylation of p53 in selenite-treated NB4 cells
Source: Cell Death Dis. 2014 Nov 27;5(11):e1542–. doi: 10.1038/cddis.2014.506 (PMC4260759; doi:10.1038/cddis.2014.506)
Supplement: Supplementary Figure Legends [file cddis2014506x4.doc]

**Supplementary Figure Legends**

Figure S1 Selenite induced ROS led to apoptosis.

Figure S2 ROS inhibited autophagy and promoted apoptosis. (a) ROS decreased the percentage of NB4 cells containing LC3 puncta. Cells were pretreated with 100 μM MnTBAP for 1 h and then exposed to 20 μM selenite for another 24 h. Cells were treated with 100 μM H_2_O_2_ as a positive control. Cells were labelled with anti-LC3 antibody and a DyLight 488-conjugated secondary antibody. The percentage of cells containing more than 8 LC3 puncta was calculated. The images were visualised using a Zeiss microscope. Bar: 10 μm. Values are shown as means ± SD (n=3), *P< 0.05. (b) ROS induced apoptosis in NB4 cells. Cells were pretreated with 100 μM MnTBAP for 1 h and then exposed to 20 μM selenite for another 24 h. Cells were treated with 100 μM H_2_O_2_ as a positive control. The percentage of apoptotic cells was measured using flow cytometry. The data are shown as means ± SD (n=3), *P< 0.05. (c) Selenite-induced ROS inhibited autophagy and promoted apoptosis in NB4 cells. Cells were pretreated with 100 μM MnTBAP for 1 h and then exposed to 20 μM selenite for another 24 h. Cells were treated with 100 μM H_2_O_2_ as a positive control. c-PARP and LC3-II were detected by western blotting.

Figure S3 The down-regulation of ULK1 promoted apoptosis in NB4 cells treated with selenite. (a) The deletion of ULK1 promoted apoptosis in NB4 cells. The percentage of apoptotic cells was measured using flow cytometry after NB4 cells were transfected with siRNA targeting ULK1. (b) The overexpression of ULK1 protected NB4 cells from apoptosis induced by selenite. The percentage of apoptotic cells was measured using flow cytometry after NB4 cells were transfected with pcDNA6-ULK1.
